# Supplementary material for: Energy efficient integrated MEMS neural network for simultaneous sensing and computing
Source: Commun Eng. 2023 Apr 29;2:19. doi: 10.1038/s44172-023-00071-6 (PMC10955817; doi:10.1038/s44172-023-00071-6)
Supplement: Supplementary file 2 — Supplemental Information [file 44172_2023_71_MOESM2_ESM.pdf]

Supplementary Information

Supplementary Notes 1: MEMS-based CTRNN Approach

Recurrent neural networks (RNN), unlike traditional feedforward neural networks (FFNN), utilize internal memory through self-feedback to preserve the sequences of input data during training. Thus, the RNNs have shown great success in sensory applications such as image, video, and audio processing, as well as in optimization, associative memories, and controls. A special yet very complex form of RNN known as a continuous-time recurrent neural network (CTRNN), uses differential equations to describe the activation level of the neurons (see Eq. S1). To perform a certain classification problem, the self-coupling and cross-coupling weights between different neurons of a CTRNN are determined through the training performed during the design phase of the network.

$$\dot{\mathbf{y}}_{\mathbf{i}} = f_i(y_1, \ldots, y_N) = \frac{1}{\tau_i}\left(-y_i + \sum_{j=1}^{N} w_{ij}\sigma(y_j) + h_i + I_i\right), i = 1,2, \ldots, N \quad \text{(Eq. S.1)}$$

where σ is an activation function, τ_i and y_i are the time constant and activation level of neuron i, respectively, w_{ij} is the connection strength between the ith neuron and the jth neuron, h is a bias term, Ii is the input to the ith neuron, and the dot operator represents the time derivative.

Supplementary Figure 1 shows schematic diagrams comparing the structure of a single feedforward neuron (FFN), a recurrent neuron (RN), and a continuous-time recurrent neuron (CTRN). The schematics show that while having self-feedback is the main difference between CT/RN and the FFN, the differential equation is the main difference between the RN and the CTRN. The first-order differential equation with a time constant τ of the CTRN model acts as a low-pass filter. Thus, the response of one CTRN may approximate the average response of many RNs. This approximation is inspired by the observation that during high-level intelligent tasks, such as classification, the activation levels of proximate neurons in the human brain can be approximated by their average. CTRNNs have recently emerged as a very attractive machine learning option as they require fewer neurons for high-level learning. For example, a CTRNN made of only four CTRNs was needed to learn eight wrist trajectories from its acceleration measurements,

[S1] where 128 RNs were needed to perform a similar task. [S2] However, CTRNNs are computationally expensive for real-time implementation as they require simultaneous solutions of highly coupled multiple differential equations. This makes them unsuitable for applications with size and power restrictions such as wearable devices. To overcome this challenge, in the presented approach, an analogy is made between the well-studied CTRNN and a network made of multiple coupled MEMS bi-stable TSs as explained next.

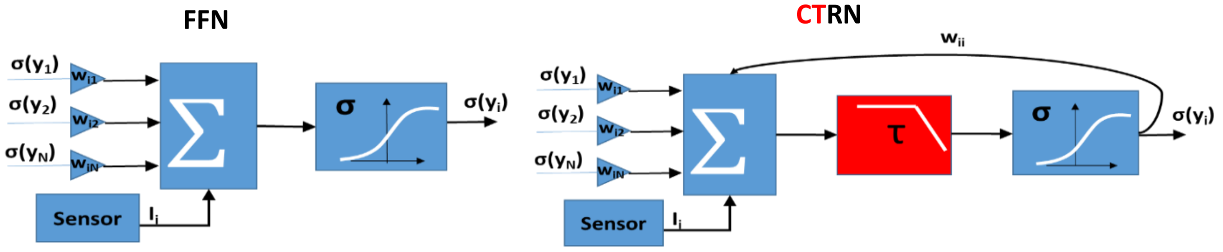

Supplementary Figure 1: The differences between FFN and CT/RN. While CTRN and RN have internal memory through self-feedback, a CTRN approximates the response of a group of RNs.

The presented small MEMS-based CTRNN network in this paper is designed to acquire one-dimensional acceleration measurements and intelligently process them to classify two different events. Supplementary Figure 2 shows a more general MEMS neural computing framework to perform any general classification tasks using two-dimensional acceleration measurements. The network has two layers, the sensing and computing layer, and the output layer. The output is added here to perform classification problems with more than two classes and is made of *O* number of typical TSs that are coupled to the sensing and computing layer. The physical characteristics (e.g., dimensions, bias voltages, etc.) of TSs in the output layer and the coupling weights with the neurons in the sensing and computing layer can be designed through 'training' to produce a binary output corresponding to a detected class of event. For example, in Supplementary Figure 2, up to eight classes can be assigned to the different binary combinations of three bits. The sensing and computing layer is made of *N* MEMS-based CTRNs. Each CTRN is made of a bi-stable TS with a relatively large proof mass to detect acceleration. The masses are oriented to move in a direction that aligns with one of the directions of the applied acceleration *a(t)*.

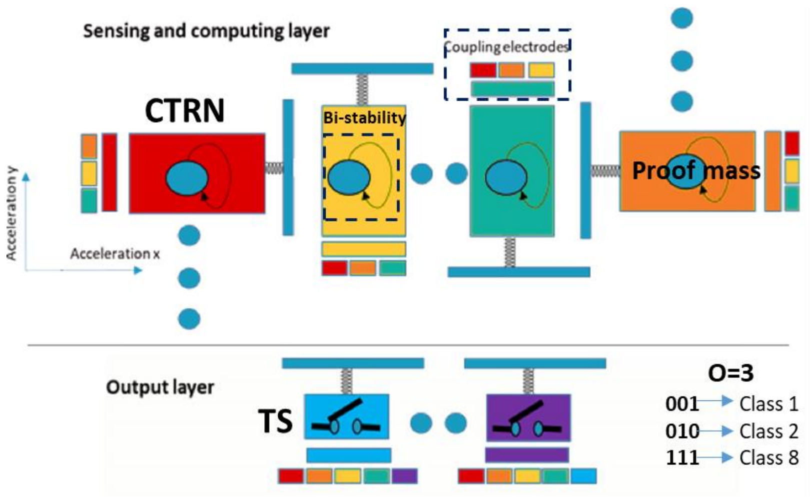

Supplementary Figure 2: *The overall proposed framework. The framework has a sensing and computing layer made of N bi-stable TSs with proof masses and an output layer made of O TSs.*

Supplementary Figure 3 schematically shows the analogy of a bi-stable TS to a single CTRN that can be integrated and coupled with other CTRNs to form the sensing and computing layer. In this analogy, the bi-stability is equivalent to the activation function (σ), the inherent TS dynamics is equivalent to the differential equation, the proof mass is equivalent to the input signal, and the electrostatic parallel plate finger array approach is equivalent to the coupling weights. While the proof mass analogy is obvious, the basis for the rest is explained next.

Activation function (bi-stability)

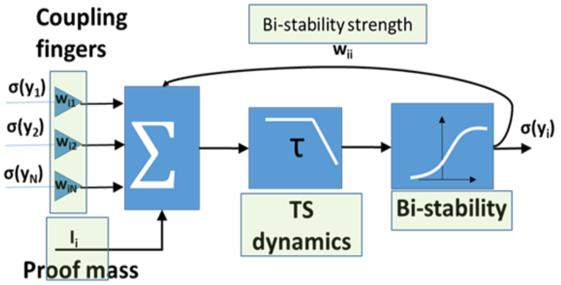

Supplementary Figure 3*: The analogy between a CTRN and a bi-stable TS with a proof mass.*

An isolated CTRN is the basic building block of a CTRNN. Its governing equation can be obtained from Eq.(S1) by setting *i* and *j* to 1 resulting in:

$$\tau\dot{\mathbf{y}}(t) = -y(t) + \ h + I(t) \ + w\,\sigma(y) \qquad \text{(Eq.S2)}$$

In this equation, the activation rate of change $\dot{y}$ is a function of sensory input I, a self-excitation feedback w, a time constant τ, and an activation function σ (t).

Using certain types of activation functions, such as the sigmoid function $\sigma(y) = \frac{1}{1+e^{-y}}$, the CTRN dynamics develop a potential energy of a double-well type (Supplementary Figure 4). To explain this behavior, the bifurcation diagram of the system fixed points is plotted. The diagram shows that the system has two stable solutions. The first occurs at lower sensory input (OFF state) and the other at higher sensory input (ON state). The separation between the two stable solutions by an unstable one creates hysteresis (bi-stability). Bi-stability will prevent the system from going to the OFF state immediately as the sensor input I fall below the value that caused the ON state. If a single bi-stable TS is realized, it will provide a better computing model compared the typical TS model that inherits the threshold-based characteristics, which ignores history values in determining the current switch status. Moreover, if the coupling between multiple bi-stable TSs can be realized to form a complete network, the network can be "trained" to perform high levels of learning such as classification.

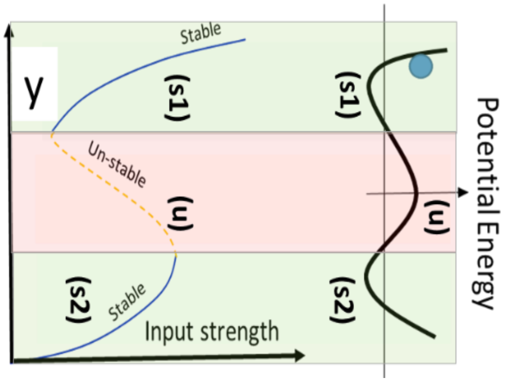

Supplementary Figure 4: CTRN dynamics with sigmoid activation function. (right): Potential well showing three local extrema, unstable (u), and stable (s1) and (s2). If the body gets close to s1 or s2, it will be attracted to them and remain within their potential well. (left) representation of the same system in a bifurcation diagram, showing the possible equilibrium positions the system may assume for a given sensory input.

While bi-stability has already been demonstrated in the literature in certain types of MEMS devices, no one has yet explored its potential use for computing like that proposed in this paper. Specifically, considering a single-degree-of-freedom model and with some valid assumptions, [S3] we have expressed the dynamic equation of a bi-stable TS with a proof mass in a form that resembles the CTRNN neuron (CTRN) as shown in Eq.(S3) and Eq.(S4). In these equations, similar terms are highlighted in the same color:

Neuron equation: $$\tau\dot{\mathbf{y}}(t) = -y(t) + h + I(t) + w\,\sigma(y) \quad \text{(Eq. S.3)}$$

Bi-stable TS equation: $$\tau\dot{\mathbf{x}}(t) = -x(t) + \mathcal{T}[V_o] + a(t) + wg(x) \quad \text{(Eq. S.4)}$$

where x is the proof mass deflection change, g is a function due to bi-stability, and $\mathcal{T}$ is a kernel function that transfers the input voltage to an electrostatic force. For parallel plate MEMS capacitors, the kernel is given by $\mathcal{T}[V] = \frac{\varepsilon A V^2}{2(d-x)^2 m}$, where m is the movable electrode mass, d, A, and V are the rest position distance, the overlap area, and the voltage difference between the two plates, respectively, and ε is the electrical permittivity.

In these equations, we describe the dynamic equation of a bi-stable TS in a way such that the $g(x)$ function resembles the σ activation function in the neuron equation. In this paper, we have utilized the Pull-in/out bi-stability to achieve the required bi-stability for computing, but as shown in Supplementary Figure 5 and explained below there are other forms of bi-stabilities that can also be utilized for such a purpose.

(3) *Pull-in/out bi-stability*: A typical electrostatic MEMS parallel plate with a thin intermediate dielectric layer h experiences a hysteresis behavior post-pull-in [S4] (Supplementary Figure 5a). In this case, the voltage needs to be reduced to a value smaller than the pull-in voltage (release voltage) to release the proof mass. The dynamic response of a parallel plate MEMS device near pull-in can be approximated to a form similar to that of Eq.(S3), where the activation-like function is given by:

$$g(x) = G(x^3 - 2(d+h)x^2) \quad \text{(Eq. S.5)}$$

where G is a function of the MEMS structure dimensions and stiffness.

(2) *Geometric nonlinearities*: An arched microbeam is a clamped-clamped microbeam with added nonlinearities to its dynamics due to an initial curvature. Bi-stability exists in an arched beam [S5] before reaching pull-in as it can jump from its original arched shape to its opposing symmetric configuration (snap-through motion), (Supplementary Figure 5. b). The dynamic response of an arched beam away from pull-in can be approximated to a form in which the activation-like function is given by:

$$g(x) = -k^* x^3 \qquad \text{(Eq. S6)}$$

where k* is the ratio between the cubic stiffness due to the initial raise and its linear stiffness.

(3) *Electrical resonance*: The last possible method relies on activating the electrical resonance of an RLC circuit, engaging a MEMS parallel plate as the capacitance in series with an external inductance (Supplementary Figure 5c). It was shown that a driving RLC circuit with a sinusoidal input voltage signal and a frequency that approaches its electric resonance frequency is another way to introduce bi-stability in MEMS before pull-in.[S6] Bi-stability is introduced through the following cycle: (1) the MEMS deflection increases due to voltage amplification at resonance, (2) the capacitance of the MEMS device changes, (3) the electrical resonance frequency shifts, (4) the voltage amplification across the MEMS parallel plate reduces, and (5) the cycle repeats until the MEMS may reach another stable position. The sigmoid-like function due to electrical resonance is as follows:

$$g(x) = \frac{V(\beta(x)-1)}{\beta(x)} \qquad \text{(Eq. S.7)}$$

where β is the electrical resonance amplification gain, which is a function of the circuit electrical damping condition, the MEMS deflection, and the difference between the input voltage frequency and the circuit resonance frequency.

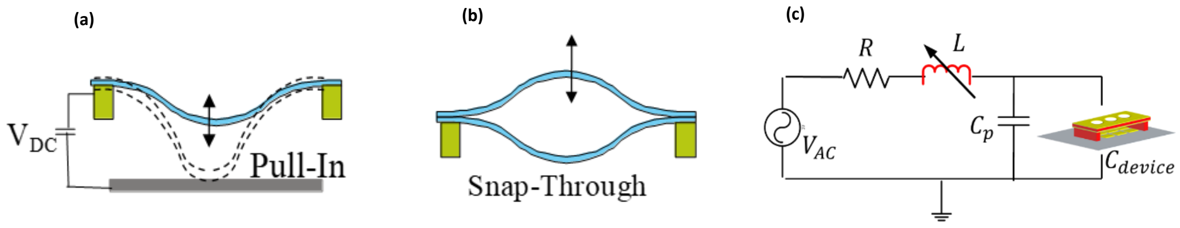

Supplementary Figure 5: Different mechanisms in electrostatic MEMS devices to introduce bistability. (a) Pull-in/out. (b) Snap-through due to initial curvature. (c) Electrical resonance activation.

Supplementary Notes 2: MEMS-based CTRNN Simulation

Here, we provide more stimulation for the MEMS neural network equation presented in the manuscript method section. In the main paper, we have presented simulation results that match the experimental data for the sit-to-stand classification problem in Fig.4. In these simulations, it is worth mentioning that hitting the MEMS network PCB by hand from the left and right sides is used to produce the NP and PN acceleration

signals that simulate the sit and stand activities. To account for the variation in the acceleration amplitude signal, a gain factor was applied in the simulation. Fig. 3a in the main paper shows the model response when where a PN (sitting activity) signal is applied. On the other hand, Fig. 3b shows the MEMS network response to the NP input acceleration (standing activity) signal. Moreover, Fig. 3c, and Fig. 3d show that when applying false signals, the MEMS network rejects those false signals. We also show in Fig.4 the effect of varying, for example, the input acceleration signal amplitude for the standing case. However, only the response of mass 1 was shown. For completeness, here in Supplementary Figure 6, we show the detailed response of each mass in the network.

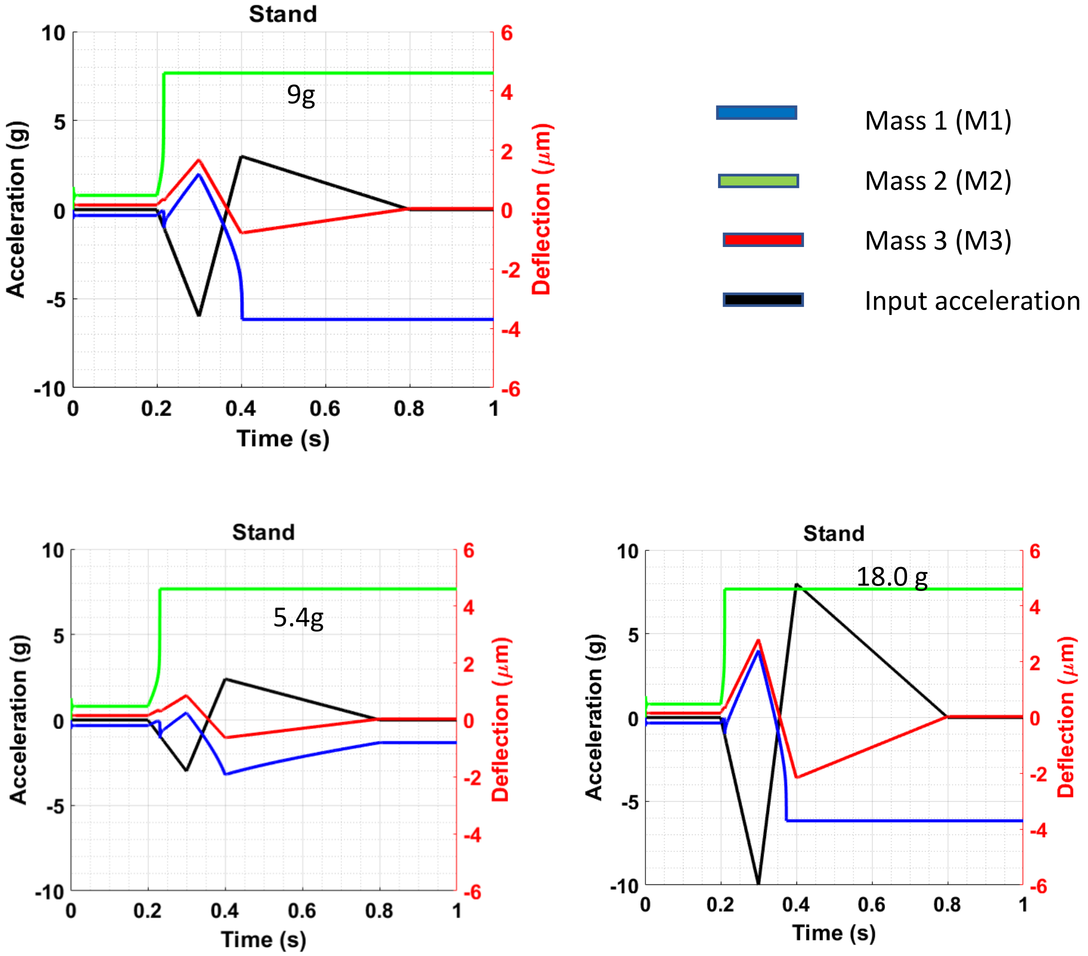

Supplementary Figure 6: Detailed simulation for all Masses responses in Fig.5 in the main manuscript for the stand case while varying the input acceleration amplitude.

Supplementary Notes 3: Energy Calculation

As the MEMS neural computing unit doesn't require any circuitry to condition and reads the acceleration measurements nor any digital computing unit to process these measurements, the DC bias voltages are the only required power source. In the current proof of concept hardware, the required DC voltage for operation is relatively high. While the device parameters can be optimized to reduce the operation voltage, due to the capacitive operation, the current hardware still requires very low power to operate. Supplementary Table 1 and Supplementary Table 2, show the calculation for the estimated energy consumption of the hardware to perform the different classification tasks.

Supplementary Table 1: Energy estimation for the MEMS neural hardware for the acceleration classification task.

Electrode	Length (µm)	Area (nm²)	Gap 1 (µm)	Gap2 (µm)	Gap #	Capacitance 1 (fF)	Capacitance2 (fF)	Vbias	Energy (pJ)
softening 1	215	10.8	3.00	18.00	8	31.7	5.29	0	0
sogtening 3	215	10.8	3.00	18.00	8	31.7	5.29	0	0
Interactings 2-1	310	15.5	7.00	25.00	6	19.6	5.49	19	27.2
Interactings 2-3	310	15.5	16.00	16.00	6	8.58	8.58	19	18.6
Comb-drives 1	6.5	0.325	4.50	3.25	51	0.639	.885	0	0
Comb-drive 3	6.5	0.325	4.50	3.25	51	0.639	.885	0	0
Softening 2	191	9.55	2.50	11.50	20	33.8	7.35	27.5	311.0
**Total Energy**							**9.9221 10^{-17} KWh**		**357 (pJ)**

Supplementary Table 2: Energy estimation for the MEMS neural hardware for the Signal classification task.

Electrode	Length (µm)	Area (nm²)	Gap 1 (µm)	Gap2 (µm)	Gap #	Capacitance 1 (fF)	Capacitance2 (fF)	Vbias	Energy (pJ)
softening 1	215	10.8	3.00	18.00	8	31.7	5.29	17	42.8
sogtening 3	215	10.8	3.00	18.00	8	31.7	5.29	24	85.3
Interactings 2-1	310	15.5	7.00	25.00	6	19.6	5.49	26	50.9
Interactings 2-3	310	15.5	16.00	16.00	6	8.58	8.58	36	66.7
Comb-drives 1	6.5	0.325	4.50	3.25	51	0.639	.885	35	47.6
Comb-drive 3	6.5	0.325	4.50	3.25	51	0.639	.885	25	24.3
Softening 2	191	9.55	2.50	11.50	20	33.8	7.35	28	323.0
**Total Energy**							**1.779 10^{-16} KWh**		**640 (pJ)**

Supplementary References

S1. Bailador, G., Roggen, D., Troster, G. and Trivino, G. (2007). "Real-time gesture recognition using Continuous Time Recurrent Neural Networks," In Proceedings of the ICST 2nd international conference on Body area networks (p. 15). ICST (Institute for Computer Sciences, Social-Informatics and Telecommunications Engineering).

S2. Shin, S., and Sung W., (2016). "Dynamic Hand Gesture Recognition for Wearable Devices with Low Complexity Recurrent Neural Networks," 2016 IEEE International Symposium on Circuits and Systems (ISCAS).

S3. Rafaiea, M., Hasan, M., and Alsaleem, F., (2019) Neuromorphic MEMS sensor network, *Appl. Phys. Lett.* vol.114, pp.163501.

S4. Younis, M. I. (2011). MEMS linear and nonlinear statics and dynamics (Vol. 20). Springer Science & Business Media.

S5. Ramini, A., Bellaredj, M. L., Al Hafiz, M. A., & Younis, M. I. (2015). Experimental investigation of snap-through motion of in-plane MEMS shallow arches under electrostatic excitation. Journal of Micromechanics and Microengineering, 26(1), 015012.

S6. Hafiz, M., Jaber, N., Kazmi, S., Hasan, M., Alsaleem, F., S.Ilyas, and Younis M., (2019) Efficient excitation of Micro/Nano Resonators and their higer order modes, *scientific reports,* vol.9 (1), pp.319.
